# Nomenclatural revision of Delphiniumsubg.Consolida (DC.) Huth (Ranunculaceae)

**DOI:** 10.3897/phytokeys.180.67126

**Published:** 2021-08-05

**Authors:** Pierre-Emmanuel DuPasquier, Véronique Andro-Durand, Lucas Batory, Wei Wang, Florian Jabbour

**Affiliations:** 1 Institut Systématique Évolution Biodiversité (ISYEB), Muséum national d’Histoire naturelle, CNRS, Sorbonne Université, EPHE, Université des Antilles, 57 rue Cuvier, CP39, 75005 Paris, France Université des Antilles Paris France; 2 Université de Neuchâtel, espace Tilo-Frey 1, 2000 Neuchâtel, Switzerland Université de Neuchâtel Neuchâtel Switzerland; 3 Direction des Collections Naturalistes – Botanique, Muséum national d’Histoire naturelle, 57 rue Cuvier, CP39, 75005 Paris, France Muséum national d’Histoire naturelle Paris France; 4 State Key Laboratory of Systematic and Evolutionary Botany, Institute of Botany, Chinese Academy of Sciences, Beijing 100093, China Institute of Botany, Chinese Academy of Sciences Beijing China; 5 University of Chinese Academy of Sciences, Beijing 100049, China University of Chinese Academy of Sciences Beijing China

**Keywords:** *
Aconitella
*, larkspur, Old World flora, paraphyly, Ranunculales

## Abstract

Recent molecular phylogenetic studies have indicated that *Aconitella* is embedded in *Consolida*, which in turn is embedded in *Delphinium*. We choose not to split the genus *Delphinium* (c. 300 species), as it is horticulturally and pharmaceutically important, by conserving a broad *Delphinium* by transferring the names from *Consolida* and *Aconitella* to *Delphinium* s.lat., and more precisely in the resurrected D.subg.Consolida. Including 58 species of *Aconitella* and *Consolida* within *Delphinium* causes fewer nomenclatural overall changes than do alternative schemes because most of the species of *Aconitella* and *Consolida* were once named under the name *Delphinium*. We present here the list of synonyms for the species once named under *Consolida* or *Aconitella* and gather the information relative to the types of these names. Two new combinations are provided, and 21 lectotypes are designated here.

## Introduction

**Figure 1.**
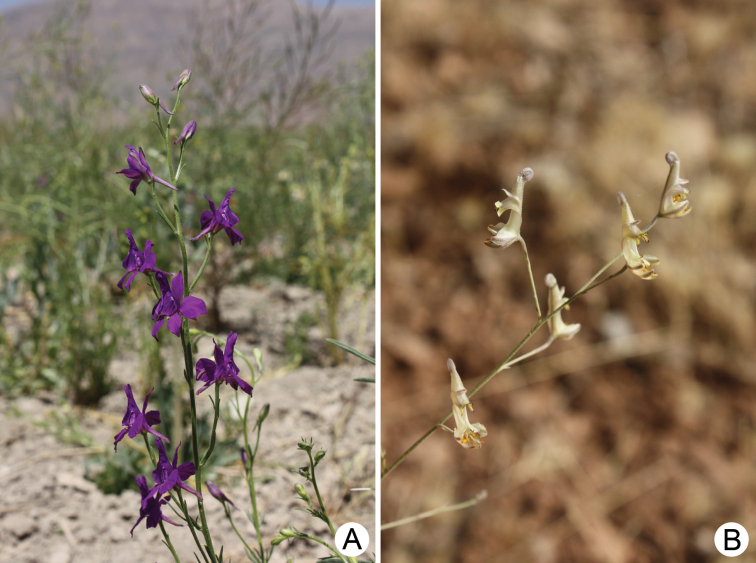
Inflorescences of Delphiniumsubg.Consolida**A***Delphiniumhispanicum* Costa **B***D.anthoroideum* Boiss. Photos: Shahin Zarre (Iran, 2011).

Different taxonomic systems based on morphological characters have led authors to treat *Consolida* J.F.Gray (including *Aconitella* Spach) (Fig. 1) and *Delphinium* L. (Ranunculaceae) as two different genera (Linnaeus 1753; Soó 1922; Davis 1965; Munz 1967a, b; Greuter and Long 1989; Chater 1993; Tamura 1993), or to consider *Consolida* as included in *Delphinium* (Candolle 1824; Boissier 1867; Huth 1895; Chowdhuri et al. 1958).

Based on a molecular phylogenetic study with a broad taxonomic sampling, Jabbour and Renner (2011) first found that *Consolida* and *Aconitella* were embedded in *Delphinium*. More precisely, *Aconitella* was nested within *Consolida*, which in turn was nested within *Delphinium*. More recent analyses confirmed this result (Jabbour and Renner 2012a; Wang et al. 2013; Xiang et al. 2017) but do not support the different subgroups (the “Untergruppen or Tribus”) previously described in *Consolida* (Huth 1895). Thus, the overemphasis on distinctive characters (see Pfeil and Crisp 2005) in *Consolida* and *Aconitella* led to recognizing a paraphyletic *Delphinium*.

As a consequence of these results, the last author previously decided (Christenhusz et al. 2018: 73) not to split the large genus *Delphinium* (c. 300 species), as it is horticulturally and pharmaceutically important (Tamura 1993), by conserving a broad *Delphinium* (see Kadereit et al. 2016) by transferring under *Delphinium* the eight species names (out of 58) of *Consolida*, all published after 1965, that were never transferred. The new combinations were: *D.arenarium* (Carlström) Jabbour, *D.coelesyriacum* (Mouterde) Jabbour, *D.kandaharicum* (Iranshahr) Jabbour, *D.lineolatum* (Hub.-Mor. & C.Simon) Jabbour, *D.lorestanicum* (Iranshahr) Jabbour, *D.samium* (P.H.Davis) Jabbour, *D.staminosum* (P.H.Davis & Sorger) Jabbour, and *D.stapfianum* (P.H.Davis & Sorger) Jabbour.

As flower morphological characters support a clade including *Consolida* and *Aconitella* (Jabbour and Renner 2012b), we treat *Consolida* as a subgenus of *Delphinium* in this article. We re-introduce here Delphiniumsubg.Consolida (DC.) Huth and list its 58 species with information relative to their typification.

## Methods

We analysed the original material cited in the protologue of each taxon and compiled the relevant synonyms. Except for rare cases, the infraspecific taxa of *Consolida* are not mentioned as taking taxonomic decisions at this taxonomic level is beyond the scope of this work.

Herbarium specimens and images of specimens from the herbaria ATHU, B, BASBG, BC, BEI, BH, BM, BP, BR, C, E, FI, FR, G, GB, GH, GOET, GZU, H, HAL, HBG, HUJ, ISL, JE, K, L, LD, LE, LI, LY, MJSD, MA, MEL, MO, NY, O, OXF, P, PAD, PH, S, TARI, UC, UPS, US, W, WAG, WRSL, and WU were examined. We studied all the available digitized specimens of the relevant collections. The following online resources were consulted: Geneva Herbaria Catalogue, JSTOR Global Plants, Kew Herbarium Catalogue, Naturalis BioPortal, Paris virtual herbarium of vascular plants, RBGE Herbarium Catalogue, Sweden’s Virtual Herbarium, the University and Jepson Herbaria, the Virtual Herbaria JACQ, and the Virtual herbarium Berolinense.

Based on the methodology of typification followed by Al-Shehbaz and Barriera (2019), we provide notes about the typification, especially if a lectotype is designated here for the first time or if earlier lectotypifications were incomplete or erroneous. In some cases and some relatively recently described species, the holotype was not found in the mentioned herbarium. When we consider that further investigations are necessary, we have decided not to designate a lectotype. Accepted names are in bold italics and listed alphabetically. The reference of cited specimens (herbarium code, and when available, the barcode) are provided. The herbarium codes follow Thiers (2018). Specimens marked ‘!’ were examined in the scope of this paper. Note that JE, WU, and W barcodes have temporary barcodes and are susceptible to change in the future (Jochen Müller, Dieter Reich, and Christian Braüchler, curators, pers. comm.).

## Results

Delphiniumsubg.Consolida consists of 58 species. Two new combinations are made, and 21 lectotypes (including three second-step lectotypifications) are designated herein.

### Typification and nomenclature

#### Delphinium subg. Consolida

Taxon classificationPlantaeRanunculalesRanunculaceae

(DC.) Huth, Bot. Jahrb. 20: 337. 1895.

DCD164CF-5BD6-5FFE-858B-0BEC431E722D

≡ Consolida S.F.Gray, Nat. Arr. Brit. Pl. 2: 711. 1821.
≡ Delphiniumsect.Consolida DC., Reg. Veg. Syst. Nat. 1: 341. 1817.
≡ Ceratosanthus Schur, Enum. Pl. Transsilv. 30. 1866. Type: Delphiniumconsolida L.
= Delphiniumsubg.Aconitella (Spach) Iranshahr, Fl. Iranica 171: 92. 1992.
≡ Aconitella Spach in Hist. Nat. Veg. 7: 358. 1839.
≡ Consolidasect.Aconitella (Spach) Tamura in Acta Phytotax. Geobot. 41: 101. 1990. Type: Delphiniumaconiti L.
= Aconitopsis Kem.-Nath. in Trudy Tbilissk. Bot. Inst. 7: 125. 1940. Type: not designated.

#### 1. Delphinium aconiti

Taxon classificationPlantaeRanunculalesRanunculaceae

L., Mant. Pl.: 77. 1767 [basionym].

1B90A341-A034-56D1-BC44-A5DB1FAB003C

≡ Consolidaaconiti (L.) Lindley in J. Roy. Hort. Soc. 6: 55. 1851.
≡ Aconitellaaconiti (L.) Soják in Folia Geobot. Phytotax. Bohem. 4: 448. 1969.
≡ Aconitopsisaconiti (L.) Kem.-Nath. in Trudy Tbilissk. Bot. Inst. 7: 125. 1940, non Aconitelladelphinioides Spach in Hist. Nat. Veg. 7: 359. 1839.
≡ Aconitummonogynum Forssk., Fl. Aegypt.-Arab. 27. 1775. Type: Turkey. “ in Dardanella”, leg. P. Forsskål (holotype not found).

##### Notes.

The description of *D.aconiti* is based on a Forsskål’s gathering made “in Dardanella”. At LINN, no Forsskål’s material was found for this taxon, whereas two gatherings, *Forsskål 913* (C10001572) and *Forsskål 914* (C10001573), are deposited at C, in the Vahl herbarium, and are likely to correspond to the type material. The typification of the Forsskål collection is complex and needs a careful examination of the letters sent by Forsskål to Linnaeus. Note that *Forsskål 914* was a priori used for the tab XIII (Vahl 1790).

#### 2. Delphinium ajacis

Taxon classificationPlantaeRanunculalesRanunculaceae

L., Sp. Pl.: 531. 1753 [basionym].

22E926B9-068C-5629-A86D-164D410E4926

≡ Consolidaajacis (L.) Schur in Verh. Mitth. Siebenbürg. Vereins Naturwiss. Hermannstadt 4: 47. 1853.
≡ Ceratosanthusajacis (L.) Schur in Enum. Pl. Transsilv. 30. 1866. Type: Herb. Burser VII(1): 83 (lectotype, designated by Molero and Blanché 1984, pg. 217: UPS image!).
= Delphinium gayanum Wilmott in J. Bot. 62: 26. 1924.
≡ Consolidagayana (Wilmott) Laínz, in Anales Inst. Forest. Invest. 1967: 6. 1967.
– Consolida ambigua auct. non Delphiniumambiguum L.

##### Notes.

*Consolidaambigua* auct. (non *D.ambiguum* L.) is a misapplied name of *D.ajacis* in most floras, such as "Flora Europaea" (Chater 1993) and "Flora of Turkey and the East Aegean Islands" (Davis 1965). For details, see Janchen (1965: 34).

#### 3. Delphinium anthoroideum

Taxon classificationPlantaeRanunculalesRanunculaceae

Boiss. in Ann. Sci. Nat. Bot. Ser. 2, 16: 369. 1841 [basionym], non sensu Boiss., Fl. Or. 1: 85. 1867.

062361F3-F3FA-50A7-877D-260F6F52BD6B

≡ Consolidaanthoroidea (Boiss.) Schrödinger in Abh. K. K. Zool.-Bot. Ges. Wien 4(5): 62. 1909.
≡ Aconitellaanthoroidea (Boiss.) Soják in Folia Geobot. Phytotax. Bohem. 4: 448. 1969.
≡ Aconitopsisanthoroidea Kem.-Nath. in Trudy Tbilissk. Bot. Inst. 7: 125. 1940. Type: “Syria”, s.d., leg. P. M. R. Aucher-Eloy 65 (lectotype, designated by Chowdhuri et al. 1958, pg. 412: G-BOIS [G00788330 image!, 2 sheets]; isolectotypes: E [E00438703 image! =photo of G00788330], G [G00390151 image!], K [K000692355 image!], P [P00195789!, P00195790!].
= Delphiniumacutilobum Turrill in Bull. Misc. Inform. Kew 1929: 223. 1929. Type: Azerbaïdjan. “near Tabriz. Yam.”, 21 Aug. 1927, leg. B. Gilliat-Smith 2086 (holotype: K [K000692358 image!]).

##### Notes.

The misinterpretation of *D.anthoroideum* by Boissier in "Flora Orientalis" (1867) is clarified by Chowdhuri et al. (1958). Among the isolectotypes of *D.anthoroideum*, only G00390151 and P00195789 bear the date “1837”.

#### 4. Delphinium arenarium

Taxon classificationPlantaeRanunculalesRanunculaceae

(A.Carlström) Jabbour in Global Fl. 4(1): 73. 2018.

67421FD2-ADD6-5F7F-8905-A3BE9D02DCA8

≡ Consolidaarenaria A.Carlström in Willdenowia 14: 16. 1984 [basionym]. Type: Greece. South Aegean: “Rodos. 2 km E of Archipolis, Stegena beach”, 13 May 1982, leg. A. Carlström 5808 (holotype: LD [LD1023446 image!]).

##### Notes.

No duplicate of the type collection was found.

#### 5. Delphinium armeniacum

Taxon classificationPlantaeRanunculalesRanunculaceae

Huth in Bot. Jahrb. Syst. 20: 380. 1895 [basionym].

3D8AB770-4EDB-52A1-80EE-9174A6DC51B1

**Figure 2.**
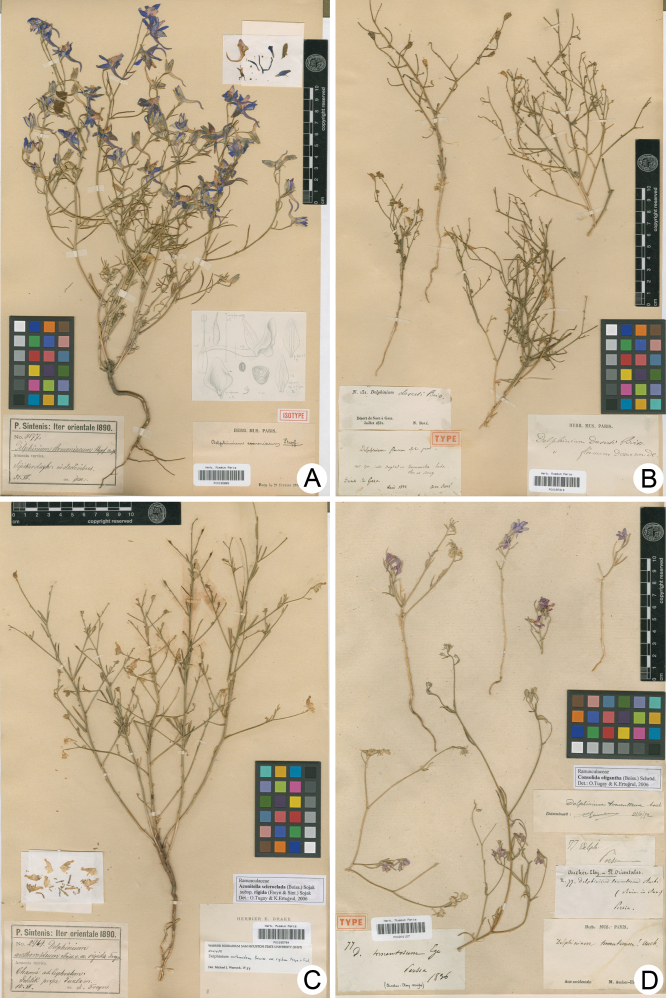
Three lectotypes (and one isolectotype) selected among the 21 lectotypes designated in this article **A** isolectotype of *Delphiniumarmeniacum* Huth (P00195865; http://coldb.mnhn.fr/catalognumber/mnhn/p/p00195865) **B** lectotype of *D.deserti* Boiss. (P00197319; http://coldb.mnhn.fr/catalognumber/mnhn/p/p00197319) **C** lectotype of DelphiniumsclerocladumBoiss.var.rigidum (Freyn & Sint.) Hossain & P.H.Davis (P00195794; http://coldb.mnhn.fr/catalognumber/mnhn/p/p00195794) **D** lectotype of *D.tomentosum* Boiss. (P00201127; http://coldb.mnhn.fr/catalognumber/mnhn/p/p00201127). All four specimens are kept at P Herbarium.

≡ Consolidaarmeniaca (Huth) Schrödinger in Abh. K. K. Zool.-Bot. Ges. Wien 4(5): 62. 1909.Type: Turkey. Erzincan: “Sipikordagh”, 30 Jul. 1890, leg. P. E. E. Sintenis 3177 (lectotype, designated here: WU [WU 109667 image!]; isolectotypes: BR [BR0000005295548 image!], G [G00390154 image!], GZU [GZU000278000 image!], JE [JE00018622 image!, JE00018623 image!], K [K000692372 image!, K000692373 image!], LD [LD1742274 image!], LE [LE01053086 image!], P [P00195865!] (Fig. 2A), PH [PH00010711 image!], WRSL [destroyed during the WWII]).

##### Notes.

Chowdhuri et al. (1958) indicated the holotype at W, but we were unable to find it. No duplicate from the above herbaria was annotated by Huth, and we choose to designate WU 109667 as the lectotype for now.

#### 6. Delphinium aucheri

Taxon classificationPlantaeRanunculalesRanunculaceae

Boiss. in Ann. Sci. Nat. Bot. Ser. 2, 16: 362. 1841 [basionym].

DC49C15B-BB69-50F9-8D05-E25DE9CAE111

≡ DelphiniumpersicumBoiss.var.aucheri (Boiss.) Boiss., Fl. Orient. 1: 77. 1867.
≡ Consolidaaucheri (Boiss.) Iranshahr in Fl. Iranica 171: 103. 1992. Type. «Persia australis», s.d., leg. P. M. R. Aucher-Eloy 4030 (holotype: P [P00198500!]; isotypes: G [G00390155 image!], P [P00198911!]).

##### Notes.

No specimen was found in Boissier’s herbarium. Boissier’s annotation on P00198500 indicates that the species description is based on that sheet, which is the holotype.

#### 7. Delphinium axilliflorum

Taxon classificationPlantaeRanunculalesRanunculaceae

DC., Syst. Nat. 1: 341. 1817 [basionym].

430913D8-9605-531A-818B-FAD5F9CC8A46

≡ Consolidaaxilliflora (DC.) Schrödinger in Abh. K. K. Zool.-Bot. Ges. Wien 4: 62. 1909. Type: «Syrie», s.d., leg. J. J. Labillardière s.n. (holotype: FI [FI056536 image!]).

##### Notes.

No duplicate of the type collection was found.

#### 8. Delphinium baluchistanicum

Taxon classificationPlantaeRanunculalesRanunculaceae

(Qureschi & Chaudhri) Jabbour & Du Pasquier comb. nov.

1DAD80EE-69BB-5ACC-9A6A-F8DFBAC5E6C0

urn:lsid:ipni.org:names:77218867-1

≡ Consolidabaluchistanica Qureschi & Chaudhri, Pakistan Syst. 2: 11. 1978 [basionym]. Type: Pakistan. Baluchistan: “Water supply station”, 23 Apr. 1977, leg. H. Mansoor & A. Maqsood 394 (holotype: ISL).

##### Notes.

When asking for an image of the holotype, the curator of ISL provided us only with an image of the specimen *Mansoor & Maqsood 395* (collected 23.04.1977), whereas the protologue indicates *Mansoor & Maqsood 394*. We could not decide whether: 1) #394 (the holotype) could not be found; or 2) #395 is the holotype, and the #394 citation in the protologue is a mistake. However, #395 seems to have been used for the drawing on plate XI in Pakistan Syst. 2. (1978).

#### 9. Delphinium barbatum

Taxon classificationPlantaeRanunculalesRanunculaceae

Bunge in Arb. Naturf. Ver. Riga 1: 126. 1847 [basionym].

912F1DDC-85C8-56CB-9790-B4D833CDC99E

≡ Consolidabarbata (Bunge) Schrödinger in Abh. K. K. Zool.-Bot. Ges. Wien 4(5): 62. 1909.
≡ Aconitellabarbata (Bunge) Soják in Folia Geobot. Phytotax. Bohem. 4: 448. 1969.
≡ Aconitopsisbarbata (Bunge) Kem.-Nath. in Trudy Tbilissk. Bot. Inst. 7: 127. 1940. Type: Kazakhstan. “jugi Karatau, ad superiorem Sarafschan”, 10 Sep. 1841, leg. A. Lehmann 38 (lectotype, designated here: P [P00197235!]; isolectotypes: LE [LE00050813 image!, LE00050814 image!]).

##### Notes.

Iranshahr (1992) indicated that the holotype is kept at P, whereas Munz (1967a) indicated that it is kept at LE, but without having seen it. We found a duplicate at P and two at LE. None of them seem to have been annotated by Bunge. The P00197235 sheet bears three specimens and two handwritten labels. These two labels are in Latin, probably from Lehmann’s hand, and correspond to the locality indicated in the protologue. They are almost identical (the left one carries “Delphinium sp ?” and “10 Sept.”, and the right one bears the collection year 1841 and no identification). Both labels were stuck on Bunge’s printed handwritten labels “Reliquiae Lehmannianae. Herb. Al. de Bunge.”, and the right one is itself stuck to another label (“Rel. Lehm. N°.38.”) written by a different hand, probably a curator of P. At LE, the labels bear the exact mention of the locality as in the protologue (in German) on a preprinted label “Alexandri Lahmann/ Reliquiae botanicae. Al. Bunge.” and one of both bears the full date.

#### 10. Delphinium brevicorne

Taxon classificationPlantaeRanunculalesRanunculaceae

Vis., Fl. Dalmat. 3: 90. 1850 [basionym].

02D2D03D-1807-5A35-BA25-8ECE49D7552B

≡ Consolidabrevicornis (Vis.) Soó in Österr. Bot. Z. 71: 245. 1922. Type. Croatia. Split-Dalmatia: “In agris circa Gelsa ins. Lesina/ as. Stalio”, s.d., Stalio s.n. (holotype: PAD [image!]).

##### Notes.

No duplicate of the type collection was found.

#### 11. Delphinium camptocarpum

Taxon classificationPlantaeRanunculalesRanunculaceae

Ledeb., Fl. Ross. 1: 58. 1841 [basionym].

CE9AEDA4-5163-558D-AA4C-F8D038E616C0

≡ Consolidacamptocarpa (Ledeb.) Nevski in Komarov, V. L., Fl. URSS 7: 106. 1937. Type: Irak. “Turcomania”, s.d., leg. G. S. Karelin, s.n. (Lectotype (first step designated by Nevski in Komarov, V. L., Fl. URSS 7: 106. 1937; second-step designated here): LE [LE00050875 image!]; isolectotypes: LE [LE00050877 image!, LE00050981 image!, LE00050986 image!).

Delphinium camptocarpum Ledeb. var. camptocarpum
≡ DelphiniumcampocarpumLedeb.var.leiocarpum Ledeb., Fl. Ross. 1: 58. 1841.

##### Notes.

When describing *Delphiniumcamptocarpum*, Ledebour quoted Karelin’s gathering “ad latus orientale maris caspii” and described two varieties (D.camptocarpumvar.dasycarpum and D.camptocarpumvar.leiocarpum) according to the presence or not of indumentum on the follicle, but without citing any material. Several of Karelin’s gatherings of *D.camptocarpum* were found at LE. Four of them belong to Ledebour’s herbarium (two can be attributed to D.camptocarpumvar.leiocarpum, but it is unclear for the other two) and can be regarded as type collection. We synonymise D.camptocarpumvar.leiocarpum under the autonym.

Nevski’s indication (1937) that the type is housed at LE can be considered a first-step lectotypification, and we designate here the specimen LE00050875 as the second-step lectotype.

#### 12. Delphinium coelesyriacum

Taxon classificationPlantaeRanunculalesRanunculaceae

(Mouterde) Jabbour in Global Fl. 4(1): 73. 2018.

44270435-6592-529B-80AF-16CF82C1558D

≡ Consolida coelesyriaca Mouterde, Nouv. Fl. Liban Syrie 2: 23. 1970 [basionym].
= Delphinium oliganthum auct. non Boiss.
= Consolida oligantha auct. non Boiss. Type: Syria. «entre Hama et Palmyre, Tell Bouada», 18 May 1857, leg. C. I. Blanche 2832 (holotype: G-BOIS [G00788352 image!]; isotype: BEI?).

##### Notes.

We did not find the duplicate at BEI, as could be expected from Mouterde’s quotation (1970).

#### 13. Delphinium consolida

Taxon classificationPlantaeRanunculalesRanunculaceae

L. Sp. Pl.: 530. 1753 [basionym].

C0069F74-CC22-5017-BCBA-8222A3BF20AB

≡ Consolida regalis S.F.Gray, Nat. Arr. Brit. Pl. 2: 711. 1821.
≡ Ceratosanthus consolida (L.) Schur in Enum. Pl. Transsilv. 30. 1866.
≡ Consolida arvensis Opiz in Seznam 32. 1852. Type: Described from Britain. Herb. Linn. No. 694.1 (lectotype, designated by Jonsell and Jarvis 1994, pg. 161: LINN [LINN-HL694-1 image!]).
= Delphinium segetum Lam. Fl. Franç. 3: 325. 1778. [nom. illeg.]
– Consolida regalis S.F.Gray subsp. consolida (L.) Gajic in Josifovic, Fl. SR Srbije 1: 230. 1970. [nom. inval].
≡ Consolida regalis S.F.Gray subsp. arvensis (Opiz) Soó, in Österr. Bot. Z. 71: 242. 1922.
≡ Delphinium consolida L. subsp. arvense (Opiz) Graebner & Graebner fil., in Asch. & Graebn., Syn. Mitteleur. Fl. 5: 671. 1929. Type: not designated.

##### Notes.

The genus *Consolida* published by Opiz (1852) is a valid but illegitimate name (Holub and Pouzar 1967).

###### Three subspecies are usually accepted under *Delphiniumconsolida*:

**13.1. DelphiniumconsolidaL.subsp.consolida**

#### 13.2. Delphinium consolida L. subsp. paniculatum

Taxon classificationPlantaeRanunculalesRanunculaceae

(Host) Busch in Kuznetzow, Fl. Cauc. Crit. 3: 44. 1902.

4D52B824-2331-57BB-979F-D96ED4EB5D81

≡ Delphinium paniculatum Host, Fl. Austriaca 2: 65. 1831.
≡ Consolida paniculata (Host) Schur. in Verh. Mitth. Siebenbürg. Vereins Naturwiss. Hermannstadt 4: 47. 1853.
≡ Consolida regalis S.F.Gray subsp. paniculata (Host) Soó in Österr. Bot. Z. 71: 243. 1922.
≡ Ceratosanthus paniculata (Host) Schur in Enum. Pl. Transsilv. 30. 1866. Type: Montenegro. “near Cattaro”, Tomasini s.n. (not found).

#### 13.3. Delphinium consolida L. subsp. divaricatum

Taxon classificationPlantaeRanunculalesRanunculaceae

(Ledeb.) A.Nyár.

57D0DCC1-8D8C-51A8-9EB9-E5AEA8F9AFDB

≡ Delphinium divaricatum Ledeb. in Eichw., Pl. Nov.: 16. 1831.
≡ Consolida divaricata (Ledeb.) Schrödinger in Abh. K. K. Zool.-Bot. Ges. Wien 4(5): 27, 62. 1909.
≡ Consolida regalis S.F.Gray subsp. divaricata (Ledeb.) Munz in J. Arnold Arbor. 48: 179. 1967.
≡ Consolida regalis S.F.Gray subsp. paniculata (Host) Soó var. divaricata (Ledeb.) P.H.Davis in Notes Roy. Bot. Gard. Edinburgh 26: 174. 1965.
≡ Ceratosanthus divaricata (Ledeb.) Schur in Enum. Pl. Transsilv. 30. 1866. Type: Russia. “In insulis ad ostium Wolgae amnis sitis, etiam ad fluvium Torrain”, Henning s.n. (holotype: LE [not seen]).

#### 14. Delphinium cornutum

Taxon classificationPlantaeRanunculalesRanunculaceae

Hossain & P.H.Davis in Notes Roy. Bot. Gard. Edinburgh 22: 424. 1958 [basionym].

7D7AD020-FC80-555C-83F7-E9E9976BECB7

≡ Consolida cornuta (Hossain & P.H.Davis) P.H.Davis in Notes Roy. Bot. Gard. Edinburgh 26: 174. 1965. Type: “Armenia”, s.d., leg. Calvert & Zohrab s.n. (holotype: E [not found]).

##### Notes.

Despite the efforts of the curator at E, the holotype was not found.

#### 15. Delphinium cruciatum

Taxon classificationPlantaeRanunculalesRanunculaceae

Hossain & P.H.Davis in Notes Roy. Bot. Gard. Edinburgh 22: 422. 1958 [basionym].

250E595E-8D2A-5430-A0DC-E7692DD29629

≡ Consolida cruciata (Hossain & P.H.Davis) P.H.Davis in Notes Roy. Bot. Gard. Edinburgh 26: 174. 1965. Type: Turkey. Adana: “Bozanti”, 1896, leg. W. Siehe 362 (holotype: E [E00438700 image!]; isotypes: B [B 10 0264874 image!], E [E00438699 image!], GH [GH00038199 image!], K [K000692438 image!], P [P00195910!]).

##### Notes.

The specimen E00438700 is explicitly designated as the holotype in the protologue, whereas the indication of collection locality and date is only found on B 10 0264874 and GH00038199.

#### 16. Delphinium deserti-syriaci

Taxon classificationPlantaeRanunculalesRanunculaceae

Zohary in Palestine J. Bot. Jerusalem Ser., 2: 155. 1941 [basionym].

3EB08F3F-0D60-5400-A14D-EA29BC21ADAD

≡ Consolida deserti-syriaci (Zohary) Munz in J. Arnold Arbor. 48: 187. 1967a.
≡ Aconitella deserti-syriaci (Zohary) Soják in Folia Geobot. Phytotax. Bohem. 4: 448. 1969. Type: Syria. “Azra to Damascus”, 16 May 1931, leg. M. Zohary s.n. (holotype: HUJ image!, fragments only).

##### Notes.

The type was partially destroyed during the war (most likely WWII) in Israel (Munz 1967a).

#### 17. Delphinium flavum

Taxon classificationPlantaeRanunculalesRanunculaceae

DC., Syst. Nat. 1: 346. 1817 [basionym].

A9E53F0C-E172-5789-8630-A2288D1B09A9

≡ Consolida flava (DC.) Schrödinger in Ann. K. K. Naturhist. Hofmus. 27: 43. 1913. Type: “de Bagdad à Kermancha”, s.d., leg. G. A. Olivier & J. G. Bruguière s.n. (lectotype, designated here: P [P00197330!]; isolectotypes: G-DC [G00200080 image!], P [P00197331!]).

Delphinium flavum DC. var. flavum
= Delphinium flavum DC. var. velutinum DC., Syst. Nat. 1: 346. 1817.
= Delphinium deserti Boiss., Fl. Orient. 1: 83. 1867.
≡ Consolida deserti (Boiss.) Munz in J. Arnold Arbor. 48: 51. 1967a. Type: «Désert de Suez à Gaza», Jul. 1832, leg. N. Bové 131 (lectotype, designated here: P [P00197319!] (Fig. 2B); isolectotypes: G [G00440765 image!, G00440766 image!], K [K000076088 image!, K000076089 image!], P [P00197320!]).

##### Notes.

In the protologue of *D.flavum*, Candolle (1817) described two varieties (var. velutinum and var. glabrum) based on the pubescence and the bracteole position on the pedicel. We synonymize here D.flavumvar.velutinum with the autonym. Candolle indicates that he saw the specimen in Olivier’s herbarium (now at P), whereas we found at G-DC a duplicate received in 1822. At P, we found two sheets of the Olivier and Bruguière’s gathering. It is not sure whether Candolle annotated these sheets. The specimen P00197330 bears the label “dans les lieux incultes steriles de Bagdad a Kermancha” and a mixture of both varieties (the specimen in the bottom right corner corresponds to D.flavumvar.glabrum). We designate as lectotype the six other specimens on this sheet.

A single and fragmentary specimen of *D.deserti*, labelled “D.deserti” by Boissier, was found in G-BOIS (G00788308) and probably came from a P duplicate. We designate as lectotype of *D.deserti* the specimen P00197319, the only one bearing a priori an annotation from Boissier.

#### 18. Delphinium glandulosum

Taxon classificationPlantaeRanunculalesRanunculaceae

Boiss. & Huet in Boiss., Diagn. Pl. Orient., Ser. 2, 5: 11. 1856 [basionym].

1E1767DD-DFED-5FA9-8ECA-1854147846A2

≡ Consolida glandulosa (Boiss. & Huet) Bornm. in Repert. Spec. Nov. Regni Veg. Beih. 89: 13. 1936. Type: Turkey. Erzurum: “in cultis Meimansour”, Aug. 1853, leg. Huet du Pavillon, A. s.n. (holotype: G-BOIS [G00788286 image!, 3 sheets]; isotypes: BM [BM000553908 image!], G [G00390160 image!, G00390161 image!], GOET [GOET009744 image!], JE [JE00018629 image!, JE00018630 image!, JE00018631 image!, JE00018632 image!], K [K000075573 image!], LE [LE00012145 image!], MO [MO-203061 image!], O [O-V2130694 image!, O-V2130695 image!], P [P00197358!, P00197359! probable], S [S07-14845 image!], UC [UC1055003 image!], WAG [WAG0004719 image!]).

##### Notes.

The description was based on the specimen in G-BOIS, a folder containing three sheets. Only this specimen was annotated by Boissier, which is, therefore, the holotype.

#### 19. Delphinium gombaultii

Taxon classificationPlantaeRanunculalesRanunculaceae

J.Thiébaut in Bull. Soc. Bot. France 81: 114. 1934 [basionym].

3F6744B6-5DEB-5FEC-8846-02274C409ECB

≡ Consolida gombaultii (J.Thiébaut) Munz in J. Arnold Arbor. 48: 175. 1967a. Type: Syria. As-Suwayda: “Djebel Druze”, 21 May 1932, leg. R. Gombault s.n. (holotype: MJSD [MJSD028148 image!]; isotypes: P [P00197360!, P00197361!]).

##### Notes.

Thiébaud (1934) based the species description on the duplicate in his herbarium, which is housed at MJSD. The duplicates at P bear the collection number *1717*, which is not indicated on the MJSD specimen.

#### 20. Delphinium halophilum

Taxon classificationPlantaeRanunculalesRanunculaceae

Huth in Bot. Jahrb. Syst. 20: 487. 1895 [basionym].

CB8D8617-1EA2-50A5-9CE7-C20CB9FA77F2

≡ Consolida halophila (Huth) Munz in J. Arnold Arbor. 48: 189. 1967a. Type: Iran. “Persia borealis. Gussediche”, 1882, leg. T. Pichler s.n. (holotype: G [G00390261 image!]; isotypes: K [K000692367 image!, K000692368 image!]).

##### Notes.

Huth (1895) based his species description solely on the unicate in the Barbey herbarium (now G).

#### 21. Delphinium hellesponticum

Taxon classificationPlantaeRanunculalesRanunculaceae

Boiss. in Ann. Sci. Nat. Bot. Ser. 2, 16: 366. 1841 [basionym].

A6603248-ED39-5B4F-B222-CDAB8FD0B935

≡ Consolida hellespontica (Boiss.) Chater in Feddes Repert. Spec. Nov. Regni Veg. 69: 55. 1964. Type: Turkey. “ad Hellespontum”, Aug. 1836, leg. P. M. R. Aucher-Eloy 67 (holotype: G-BOIS [G00788295 image!, 2 sheets]; isotypes: G [G00440745 image!], K [K000075580 image!], P [P00197463!, P00201125!]).
= Delphinium macedonicum Halácsy & Charrel in Charrel, Géogr. Bot. Salonique: 8. 1892.
≡ Consolida macedonica (Halácsy & Charrel) Soó in Österr. Bot. Z. 71: 245. 1922.
≡ Delphinium hellesponticum subsp. macedonicum (Halácsy & Charrel) Hossain & P.H.Davis in Notes Roy. Bot. Gard. Edinb. 22: 419. 1958.
≡ Consolida hellespontica subsp. macedonica (Halácsy & Charrel) Chater in Feddes Repert. Spec. Nov. Regni Veg. 69: 55. 1964. Type: Greece. Thessaloniki: “Kiel tépé [probably Profitis Ilias, SE of Chortiatis fide Strid, 2002]”, s.d., A.-u.-R. Nadji s.n. (holotype: WU (WU033827 image!), isotype: P [P02500001!, P02840902!])
= Delphinium paphlagonicum Huth in Bull. Herb. Boissier 1: 328. 1893.
≡ Delphinium olopetalum Boiss. var. paphlagonicum (Huth) Huth, in Engler, Bot. Jahrb. 20: 381. 1895. Type: Turkey. Kastamonu: “Paphlagonia: Wilajet Kastambuli, Tossia”, 7 Aug. 1892, leg. P. E. E. Sintenis 4547 (lectotype, designated here: LD [LD1742402 image!], isolectotypes: B [B 10 0295663 image!, B 10 0264875 image!], BH image!, GZU [GZU000279189 image!], HAL [HAL0062300 image!], HBG [HBG508807 image!],, P [P00197464!, P00198840!], US [US00103588 image!]).
= Delphinium hellesponticum subsp. aintabense Hossain & P.H.Davis in Notes Roy. Bot. Gard. Edinburgh 22: 420. 1958. Type: Turkey. “Aintab [Gaziantep]”, Jun. 1889, leg. G. E. Post, s.n. (holotype: BM [BM013718242 image!]).
= Delphinium campylopodum Stapf in Denkschr. Acad. Wiss. Wien, Math. Naturw. Kl. 51: 358. 1886.
≡ Delphinium hellesponticum Boiss. subsp. campylopodon (Stapf) Hossain & P.H.Davis in Notes Roy. Bot. Gard. Edinburgh 22: 419. 1958. Type: “Owadjik”, 1 Aug. 1882, leg. F. Luschan s.n. (lectotype, designated by Chowdhuri et al. 1958, pg. 419): WU [WU0072944 image!]).

##### Notes.

Boissier based the species description of *Delphiniumhellesponticum* on the G-BOIS specimens, which is a folder containing two sheets. Only the K duplicate bears a date (“Aug. 1836”). Among the duplicates of *Delphiniummacedonicum*, only P02840902 bears a date (27 avril 1892) and the collection number *37*. Huth based his description of *Delphiniumpaphlagonicum* on the *Sintenis 4547* specimen in his herbarium. The duplicates found in the different herbaria bear different dates, and only LD1742402 (with the date “7 Aug. 1892”), belonging to the Sintenis herbarium, was annotated by Huth, and we treat it as a lectotype. All isotypes indicate herein bear that date.

#### 22. Delphinium hispanicum

Taxon classificationPlantaeRanunculalesRanunculaceae

Costa in Anales Soc. Esp. Hist. Nat. 2: 27. 1873 [basionym].

0F4072C5-FA71-5BB7-B462-D15510B9E289

≡ Consolida hispanica (Costa) Greuter & Burdet in Willdenowia 19: 43. 1989.
≡ Consolida orientalis (Gay) Schrödinger subsp. hispanica (Willk.) P.W.Ball & Heywood in Feddes Repert. Spec. Nov. Regni Veg. 66: 151. 1962. Type: Spain. «Linares más arriba de la región de la vid.», 1864, leg. Vivas s.n. (lectotype, designated by Blanché and Simón 2000, pg. 304): BC image!, specimen on the left side).
= Delphinium orientale Gay in Actes Soc. Linn. Bordeaux 11: 182. 1840 [nom. illeg.]
≡ Consolida orientalis (Gay) Schrödinger in Abh. K. K. Zool.-Bot. Ges. Wien 4(5): 27, 62. 1909. Type: not designated.
= Delphinium bithynicum Griseb. in Spic. Fl. Rumel. 1: 320. 1843 [basionym]. Type: Turkey. “Bolu”, s.d., leg. F. Pestalozza s.n. (holotype: GOET [GOET009749 image!]).

##### Notes.

*Delphiniumorientale* Gay was misapplied instead of *D.hispanicum* in most Floras e.g. "Flora Orientalis" (Boissier 1867), "Flore de l’Afrique du Nord" (Maire 1952), "Flora of Syria, Palestine and Sinaï" (Post 1932), "Flora of Turkey and the East Aegean Islands" (Davis 1965). See Greuter and Raus (1989) for a discussion of the nomenclature.

#### 23. Delphinium hohenackeri

Taxon classificationPlantaeRanunculalesRanunculaceae

Boiss., Fl. Orient. 1: 85. 1867 [basionym].

EE7E5B49-FA83-5CED-90A3-037098167FCA

≡ Consolida hohenackeri (Boiss.) Grossh., Fl. Kavkaza 2: 101. 1930.
≡ Aconitella hohenac keri (Boiss.) Soják in Folia Geobot. Phytotax. Bohem. 4: 448. 1969.
≡ Aconitopsis hohenackeri (Boiss.) Kem.-Nath. in Trudy Tbilissk. Bot. Inst. 7: 127. 1940. Type: Turkey. Bayburt: “Baibout Mt. du Tchorok coteaux arides”, 12 Jul. 1862, leg. E. Bourgeau 21 (lectotype (first step designated by Chowdhuri et al. 1958, pg. 415; second-step designated here): G-BOIS [G00788345 image!]; isolectotypes: E [E00438706 image!], JE [JE00018599 image!], LY [LY0042520 image!], P [P04023369!, P00195762!, P00195791!, P00195763!, P00195764!], UC [UC1055009 image!]).

##### Notes.

The designation by Chowdhuri et al. (1958) of *Bourgeau 7* at K as the lectotype is corrected herein as a second-step typification from K (where no Bourgeau’s gathering of *D.hohenackeri* was found) to G-BOIS. However, the only Bourgeau’s gathering of *D.hohenackeri* at G-BOIS bears the collection number *21*. In contrast, *Bourgeau 7* is found at E, JE, LY, P, and UC (*sub D.anthoroideum*), usually on printed Bourgeau’s collection labels.

#### 24. Delphinium incanum

Taxon classificationPlantaeRanunculalesRanunculaceae

E.D.Clarke, Travel 2(1): 451. 1812 [basionym].

91CD79D6-F2A7-559A-B622-C2697588A32F

≡ Consolida incana (E.D.Clarke) Munz in J. Arnold Arbor. 48: 181. 1967a. Type: Israel: “Migdal”, 13 Jun. 1942, leg. P. H. Davis 4819 (neotype, designated by Munz 1967a, pg. 181: BM [BM013718647 image!]; isoneotype: E [E00438698 image!]).
= Delphinium exsertum DC., Syst. Nat. 1: 345. 1817 [basionym]. Type: sine loco, Labillardière, J. J. (holotype: FI [FI058566 image!; isotype: FI [FI058565 image!]).
= Delphinium rigidum DC., Syst. Nat. 1: 344. 1817.
≡ Consolida rigida (DC.) Bornm. in Beih. Bot. Bot. Centralbl., Abt. 2. 31: 181. 1914. Type: “Syria”, s.d., leg. J. J. Labillardière s.n. (holotype: G [G00390153 image!]).

##### Notes.

Munz (1967a) designated an isoneotype for *D.incanum* at K, which was not found. At FI, the specimen FI058564 could correspond to a duplicate of the type material of *D.rigidum*.

#### 25. Delphinium intrincatum

Taxon classificationPlantaeRanunculalesRanunculaceae

Pau in Trab. Mus. Cienc. Nat. Ser. Bot. 14: 12. 1918 [basionym].

D5D3D349-8240-5FE6-8E8E-66099F529B56

≡ Consolida teheranica (Boiss.) Rech. f. var. intrincata (Pau) Parsa, Fl. Iran 2: 326. 1986.
≡ Aconitella intrincata (Pau) C.Blanché & J.Molero in Bot. J. Linn. Soc. 113: 127. 1993. Type: Iran. «Kouh-Cherri (Alto Karum)», 23 Jul. 1899, leg. M. de la Escalera s.n. (lectotype, designated by Blanché and Molero 1993, pg. 127: MA [MA39257 image!]).

##### Notes.

No duplicate of the type collection was found in the different herbaria consulted.

#### 26. Delphinium kabulianum

Taxon classificationPlantaeRanunculalesRanunculaceae

Akhtar in Bull. Misc. Inform. Kew 1938: 86. 1938 [basionym].

0A5E0319-038A-58F1-94EC-8DDFC4F15F85

≡ Consolida kabuliana (Akhtar) Iranshahr in Fl. Iranica 171: 102. 1992.
≡ Consolida stocksiana (Boiss.) Nevski var. kabuliana (Akhtar) Tamura in Kitamura, Fl. Afghan. 124. 1960. Type: Afghanistan. “near Kabul”, 23 Aug. 1937, leg. S. A. Akthar (holotype: K [K000692442 image!]).

##### Notes.

No duplicate of the type collection was found in the different herbaria consulted.

#### 27. Delphinium kandaharicum

Taxon classificationPlantaeRanunculalesRanunculaceae

(Iranshahr) Jabbour in Global Fl. 4(1): 73. 2018.

DE487E51-0A85-50AD-B2FE-C95AC2B21681

≡ Consolida kandaharica Iranshahr in Pl. Syst. Evol. 155: 56. 1987 [basionym]. Type: Afghanistan. Kandahar: “versus lacum artificiale / Arghandab Reservoir”, 22–23 May 1967, leg. K. H. Rechinger 34869 (holotype: W [W1984-0011834 image!]).

##### Notes.

No duplicate of the type collection was found in the different herbaria consulted.

#### 28. Delphinium leptocarpum

Taxon classificationPlantaeRanunculalesRanunculaceae

(Nevski) Nevski in Fl. URSS 7: 110. 1937

D3307E49-752F-58E5-986F-154924C1E2B0

≡ Consolida leptocarpa Nevski in Acta Inst. Bot. Acad. Sci. URSS 4: 296. 1937 [basionym]. Type: «Ak-Davan», 21 Jun. 1931, S. A. Nevski 364 (holotype: LE [LE00050815 image!]; isotype: K [K000692381 image!]).

##### Notes.

Although Nevski annotated both duplicates at LE and K, the latter is clearly labelled “Dupla”, and we considered LE00050815 as the holotype.

#### 29. Delphinium linarioides

Taxon classificationPlantaeRanunculalesRanunculaceae

Boiss. in Ann. Sci. Nat. Bot. ser. 2, 16: 368. 1841 [basionym].

0CF0FDDB-5543-5DDC-9163-2B7278C2996E

≡ Consolida linarioides (Boiss.) Munz in J. Arnold Arbor. 48: 191. 1967a. Type: Iran. “Ispahan”, s.d., leg. P. M. R. Aucher-Eloy 4029 (lectotype, designated here: G-BOIS [G00788305 image!]; isolectotypes: G [G00440762 image!], K [K000692379 image!], P [P00198677!, P00198678!]).

##### Notes.

Boissier’s annotation is found on P00198677 and G00788305, indicating Boissier based the species description on these duplicates. Therefore, the lectotypification is justified.

#### 30. Delphinium lineolatum

Taxon classificationPlantaeRanunculalesRanunculaceae

(Huber-Morath & C.Simon) Jabbour in Global Fl. 4(1): 73. 2018.

0A21A1ED-2B89-506A-B068-35AB18E34B46

≡ Consolida lineolata Huber-Morath & C.Simon in Bauhinia 6: 285. 1978 [basionym]. Type: Turkey. Ermenek: “41 km sw Mut”, 13 Jul. 1976, leg. M. Nydegger 11138 (holotype: BASBG [BASBG-00000081 image!]; isotype: G [G00440764 image!, 3 sheets], GOET [GOET009747 image!]).

##### Notes.

The protologue and G00440764 indicate erroneously that the collection number is *1138* when it is *11138*.

#### 31. Delphinium lorestanicum

Taxon classificationPlantaeRanunculalesRanunculaceae

(Iranshahr) Jabbour in Global Fl. 4(1): 73. 2018.

62B07E89-8DB8-5A3C-8DDF-BC4D56363319

≡ Consolida lorestanica Iranshahr in Pl. Syst. Evol. 155: 55. 1987 [basionym]. Type: Iran. Lorestan: “10–20 km on road from Aligodarz to Shoulabad”, 2 July 1977, leg. Runemark & Lazari 26530 (holotype: TARI image!).

##### Notes.

No duplicate of the type collection was found in the different herbaria consulted.

#### 32. Delphinium mauritanicum

Taxon classificationPlantaeRanunculalesRanunculaceae

Cosson in Bull. Soc. Bot. France 27: 68. 1880 [basionym].

4334D2C9-3201-5776-9E41-C3A07017FF97

≡ Consolida mauritanica (Cosson) Munz in J. Arnold Arbor. 48: 48. 1967b. Type: Algeria. Oran: “Champs incultes à Lalla-Maghrnia”, 24 May 1856, leg. E. Bourgeau s.n. (lectotype, designated here: P [P02336111!]; isolectotypes: K [K001394825 image!], P [P02379147!]).

##### Notes.

In his protologue, Cosson quotes several syntypes. Munz (1967b) designated the gathering *Bourgeau* at P as type. We found two duplicates at P, of which only P02336111 bears an annotation from Cosson.

#### 33. Delphinium oliverianum

Taxon classificationPlantaeRanunculalesRanunculaceae

DC., Syst. Nat. 1: 341. 1817 [basionym].

EB43CF25-D5AC-5B5F-BA57-C80736D68BB5

≡ Consolida oliveriana (DC.) Schrödinger in Abh. K. K. Zool.-Bot. Ges. Wien 4(5): 62. 1909. Type: Irak. «de Bagdad à Kermachan», s.d., leg. G. A. Olivier & J. G. Bruguière s.n. (holotype: P [P00198747!]).

##### Notes.

No specimen was found at G. Candolle (1817) based on the species description on the unicate in Olivier’s herbarium, which is housed at P.

#### 34. Delphinium olopetalum

Taxon classificationPlantaeRanunculalesRanunculaceae

Boiss. in Ann. Sci. Nat. Bot. Ser. 2, 16: 364. 1841 [basionym], non sensu Hayek in Repert. Spec. Nov. Regni Veg. Beih. 30(1): 313. 1924.

830FEBFC-285F-54A2-8968-34BE714FCF32

≡ Consolida armeniaca (Huth) Schrödinger var. olopetala (Boiss.) Parsa, Fl. Iran. 2: 316. 1986. Type: “Perse ?”, 1837, leg. P. M. R. Aucher-Eloy, s.n. (holotype: P [P00198568!]).

##### Notes.

No specimen was found in Boissier’s herbarium. Boissier’s annotation on P00198568 indicates that the species description is based on this unicate (“exemplar unicum”), which is the holotype. Boissier (1841) indicated that the collection was mixed with *D.tomentosum*, which is no longer the case.

#### 35. Delphinium paradoxum

Taxon classificationPlantaeRanunculalesRanunculaceae

Bunge in Arbeiten Naturf. Vereins Riga 1: 124. 1847 [basionym].

0414D6A9-1A30-5DDD-B9D1-2AF0BC77F64D

≡ Consolida paradoxa (Bunge) Nevski in Komarov, V. L., Fl. URSS 7: 113. 1937.
≡ Consolida rugulosa (Boiss.) Schrödinger f. paradoxa (Bunge) Iranshahr in Fl. Iranica 171: 105. 1992. Type: Iran. “Djan-Darja”, 3 May 1842, leg. A. Lehman 36 (lectotype, designated here: P00198841, isolectotypes: BM [BM000946072 image! =photo of LE], GH [GH00038197 image!], H [H1506244 image!], K [K000692370 image!], LE).

##### Notes.

When describing new species, Bunge usually annotated the specimens cited with “mihi” or “m.” behind the species name. Unfortunately, we did not find any annotation from Bunge on the different duplicates. P00198841 bears ten individuals and three different preprinted Lehmann’s collection labels, all corresponding to collection number 36. One of them is from Lehmann’s hand with the locality (in Latin) and the date indicated in the protologue. No date is indicated on the other duplicates. LE (photo seen at BM) bears a label with the locality translation in German. After an investigation by the curator, the LE specimen was not found.

#### 36. Delphinium persicum

Taxon classificationPlantaeRanunculalesRanunculaceae

Boiss. in Ann. Sci. Nat. Bot. Ser. 2, 16: 362. 1841 [basionym].

67C120B9-154F-5E44-B2A7-310645BE2A07

≡ Consolida persica (Boiss.) Schrödinger in Abh. K. K. Zool.-Bot. Ges. Wien 4(5): 17. 1909. Type: Iran. “Persia Circa Amadan”, 1837, leg. P. M. R. Aucher-Eloy 78 (holotype: G-BOIS [G00150139 image!]; isotypes: G [G00192123 image!, G00192122 image!]; K [K000692364 image!]; P [P00198487!, P00198912!]).

##### Notes.

Boissier based the species description on the duplicate in G-BOIS. The date “1837” is only indicated on the isotypes housed at G and P.

#### 37. Delphinium phrygium

Taxon classificationPlantaeRanunculalesRanunculaceae

Boiss. in Ann. Sci. Nat. Bot. Ser. 2, 16: 363. 1841 [basionym].

FA467E7A-F441-56D4-80A8-F2D8529AEE26

≡ Delphinium ajacis L. var. phrygium Fin. & Gagnep. in Bull. Soc. Bot. France 51: 467. 1904.
≡ Consolida phrygia (Boiss.) Soó in Österr. Bot. Z. 71: 245. 1922.
≡ Consolida orientalis (Gay) Schrödinger subsp. phrygia (Boiss.) Chater in Feddes Repert. Spec. Nov. Regni Veg. 68: 193. 1963. Type: Turkey. “ in Phrygia”, 1831, leg. P. M. R. Aucher-Eloy 71 (holotype: P [P00198754!]; isotype: G-BOIS [G00789467 image! fragments only]).

##### Notes.

Duplicate found in G-BOIS correspond to fragments taken from P00198754, which bears an original collection label, and was examined and annotated by Boissier for the species description.

**37.1. DelphiniumphrygiumBoiss.subsp.phrygium**

#### 37.2. Delphinium phrygium Boiss. subsp. thessalonicum

Taxon classificationPlantaeRanunculalesRanunculaceae

(Soó) Jabbour & Du Pasquier comb. nov.

1FBDFD29-E310-5976-A8B3-27213524DFE8

urn:lsid:ipni.org:names:77218868-1

≡ Consolida orientalis (Gay) Schrödinger var. thessalonica Soó in Österr. Bot. Z. 71: 239. 1922 [basionym].
≡ Consolida phrygia (Boiss.) Soó subsp. thessalonica (Soó) P.H.Davis in Notes Roy. Bot. Gard. Edinburgh 26: 174. 1965. Type: Greece. Thessalia: “Kalampaka”, 4 Jun. 1896, leg. P. E. E. Sintenis 579 (holotype: not found; isotypes: E [E00346595 image!], LD [LD1742978 image!], P [P02500761!, P02574100!, P02819490!, P02819491!]).

##### Notes.

Describing the subspecies, Soó indicated two herbaria (the herbarium of the National Museum of Hungary and Borbás’ herbarium), which are at BP now. However, after an investigation by the curators, no type specimen was found. None of the isotypes cited herein are annotated by Soó. More investigation is needed to argue that the BP specimens are lost before designating any lectotype.

#### 38. Delphinium pubescens

Taxon classificationPlantaeRanunculalesRanunculaceae

DC. in Lam. & DC., Fl. Franç., Ed. 3 5: 641. 1815 [basionym].

0DDC8BF5-842D-5884-A7ED-A56F6EDF0F84

≡ Consolida pubescens (DC.) Soó in Österr. Bot. Z. 71: 241. 1922.
≡ Delphinium consolida L. subsp. pubescens (DC.) Nyman Consp. Fl. Eur. 21. 1878. Type: France. Occitanie: «lieux cultivés près Fontfroide», 12 Jun. 1807, A. P. Candolle s.n. (lectotype, designated here: G00131934 image!).
= Delphinium loscosii Costa in Anales Soc. Esp. Hist. Nat. 2: 26. 1873.
≡ Delphinium pubescens DC. subsp. loscosii (Costa) Graeber & Graeber fil., in Asch. & Graebn., Syn. Mitteleur. Fl., 5(2): 676. 1929.
≡ Delphinium consolida L. var. loscosii (Costa) Pau, in Not. Bot. Fl. Españ. 4: 12. 1891.
≡ Consolida loscosii (Costa) Holub., in Novit. Bot. Delect. Seminum Horti Bot. Univ. Carol. Prag. 1960: 4. 1960.
≡ Consolida pubescens (DC.) Soó subsp. loscosii (Costa) Soó, in Österr. Bot. Z. 71: 241. 1922.
≡ Consolida pubescens (DC.) Soó var. loscosii (Costa) P.W.Ball & Heywood, in Feddes Repert. Spec. Nov. Regni Veg. 66: 151. 1962. Type: Spain. “in Aragonia australis pratis arvisque quoque in Catalaunia”, s.d., leg. A. C. Costa s.n. (lectotype, designated by Blanché and Simon 2000, pg. 307: BC [BC-975765 image!]).

##### Notes.

Candolle did not mention any specimen in his protologue when he described *D.pubescens*. Although some original material of the "Flore Française" can be at MPU or P (Stafleu 1967), where Candolle worked before moving to Geneva, we found a Candolle’s gathering dated 1807 at G in the prodromus herbarium, that we designate as the lectotype.

#### 39. Delphinium pusillum

Taxon classificationPlantaeRanunculalesRanunculaceae

Labill., Icon. Pl. Syr. 4: 5. 1812 [basionym].

469E5B33-9E9E-51AD-9D2C-50BCF83CA0BE

≡ Consolida pusilla (Labill.) Schrödinger in Abh. K. K. Zool.-Bot. Ges. Wien 4(5): 62. 1909. Type: Syria. “Djebel Cher”, s.d., leg. J. J. Labillardière s.n. (holotype: FI [FI005591 image!).
= Delphinium pygmaeum Poiret in Lam., Encycl. Suppl. 2: 458. 1812.
≡ Consolida pygmaea (Poiret) Schrödinger in Ann. K. K. Naturhist. Hofmus. 27: 43. 1913. Type: “Syrie”, s.d., leg. J. J. H. Labillardière s.n. (holotype: FI [FI005590 image!]; isotype: P [P04023292!]).
= Delphinium oliganthum Boiss. Fl. Orient. 1: 80. 1867.
≡ Consolida oligantha (Boiss.) Schrödinger in Ann. K. K. Naturhist. Hofmus. 27: 43. 1913.
≡ Consolida tomentosa (Boiss.) Schröd. subsp. oligantha (Boiss.) P.H.Davis in Notes Roy. Bot. Gard. Edinburgh 26: 175. 1965. Type: Syria. “In agris apris: p. Assy. p Aintab”, 27 Jun. 1865, leg. H. C. Haussknecht s.n. (lectotype (first step designated by Chowdhuri et al. 1958, pg. 417, second-step designated here): G [G00788351a image!, not G00788351]; isotypes: G [G00390259 image!, specimen in the middle only], K [K000075574 image!, two specimens annotated as “3”], P [P00198731!, P00550831!, P00198733!, P00198737!]).

##### Notes.

The holotype of *D.pusillum* bears a handwritten protologue by Labillardière. Munz (1967a) indicated that he saw an isotype of *D.pusillum* at K (obviously K000692380), but it is not clear whether this specimen belongs to the type collection or not.

As explained by Chowdhuri et al. (1958), when dealing with *D.oliganthum*, the Haussknecht’s gathering housed at G and K is a mixed collection of *D.hellesponticum* and *D.oliganthum*. In the Boissier herbarium, the folder of *D.oliganthum* contains two sheets, one with *D.hellesponticum* (G00788351), which bears the original label, and the other one with *D.oliganthum* (G00788351a), both annotated in 1956 by M. Hossain, one of the co-authors with P. K. Chowdhuri and P. H. Davis. At G, K and P duplicates also contain both species. We complete the first step of lectotypification by Chowdhuri et al. (1958), who indicated G and K simultaneously in designating the specimen on the sheet G00788351a at G-BOIS as the second-step lectotype.

#### 40. Delphinium raveyi

Taxon classificationPlantaeRanunculalesRanunculaceae

Boiss., Diagn. Pl. Orient. 1: 66. 1843 [basionym].

A3EF019D-FE64-5562-BCEC-C63F87DEA216

≡ Consolida raveyi (Boiss.) Schrödinger in Abh. K. K. Zool.-Bot. Ges. Wien 4(5): 62. 1909. Type: Turkey. Aydin: “in arvis Cariae ad Geyra”, Jun. 1842, leg. P. E. Boissier s.n. (lectotype, designated here: G-BOIS [G00330114 image!, 5 sheets]; isolectotypes: BM [BM000553909 image!], G [G00390157 image!, 2 sheets, G00390156 image!], GOET [GOET009748 image!], JE [JE00018604 image!, JE00018605 image!, JE00018606 image!], K [K000692363 image!, K000692382 image!], MEL [MEL2409727 image!], NY [NY00353414 image!, NY00353415 image!], P [P00344101!, P00198977!, P00198978!, P00198979!], S [S07-15317 image!], US [US00409759 image!]).

##### Notes.

Boissier annotated P00198978 and, therefore, we designate as lectotype the duplicate, including five sheets, in his herbarium.

#### 41. Delphinium rugulosum

Taxon classificationPlantaeRanunculalesRanunculaceae

Boiss. in Ann. Sci. Nat. Bot. Ser. 2, 16: 361. 1841 [basionym].

28669C15-7B9F-5569-8522-99BDC639B88A

≡ Delphinium camptocarpum Fisch. & C.A.Meyer var. rugulosum (Boiss.) Bunge in Arb. Naturf. Ver. Riga 1: 126. 1848.
≡ Consolida rugulosa (Boiss.) Schrödinger in Ann. K. K. Naturhist. Hofmus. 27: 43. 1913. Type: Iran.”ad lacum Ourmiah”, s.d., leg. P. M. R. Aucher-Eloy 4028 (holotype: G-BOIS [G00150138 image!]; isotypes: BM [BM000570952 image!], G [G00192124 image!], K [K000692365 image!, K000692366 image!], P [P00198502!, P00198503!, P00198504!, P00198505!]).

##### Notes.

Boissier based the species description on the unicate at G-BOIS.

#### 42. Delphinium saccatum

Taxon classificationPlantaeRanunculalesRanunculaceae

Huth in Bull. Herb. Boissier 1: 328. 1893 [basionym].

5D664370-6BBE-5167-80B2-761BC1A01A30

≡ Consolida saccata (Huth) P.H.Davis in Notes Roy. Bot. Gard. Edinburgh 26: 173. 1965.
≡ Aconitella saccata (Huth) Soják in Folia Geobot. Phytotax. Bohem. 4: 448. 1969.
≡ Aconitopsis saccata (Huth) Kem.-Nath. in Trudy Tbilissk. Bot. Inst. 7: 127. 1939. Type: Turkey. “Mardin: Rischemil, in lapidosis”, 28 Jun. 1888, leg. P. E. E. Sintenis 1186 (lectotype, designated here: LD [LD1016965 image!]; isolectotypes: BR [BR0000005295852 image!], E [E00438701 image!], G [G00440763 image!], JE [JE00018615 image!, JE00018616 image!, JE00018617 image!, JE00018618 image!], K [K000692359 image!, K000692360 image!], LD [LD1017157 image!], MO [MO-2196034], P [P00198522!, P00198523!, P00198524!], PH [PH00010734 image!], S [S07-15323 image!]).

##### Notes.

Huth (1893) saw the *Sintenis 1186* specimen at B (but destroyed during WWII) and in his personal herbarium. Among the duplicates, we found specimens annotated by Huth at K (K000692359) and LD (LD1016965). Therefore, their lectotypification was needed.

#### 43. Delphinium samium

Taxon classificationPlantaeRanunculalesRanunculaceae

(P.H.Davis) Jabbour in Global Fl. 4(1): 73. 2018.

0922B384-17E7-559D-A3D1-195BC9C5E63C

≡ Consolida samia P.H.Davis in Notes Roy. Bot. Gard. Edinburgh 26: 172. 1965 [basionym]. Type: Greece, North Aegean. “Samos. SW-slope of Mt. Kerki”, 26 May 1963, leg. H. Runemark & S. E. Snogerup 19592 (holotype: LD [LD1023318 image!]; isotype: E [E00202626 image!]). Davis (1965) indicated that the holotype was kept at LD.

#### 44. Delphinium schlagintweitii

Taxon classificationPlantaeRanunculalesRanunculaceae

Huth in Bull. Herb. Boiss. 1: 329. 1893 [basionym].

9684F7D2-CB22-5A03-8546-AA743E79A029

≡ Consolida schlagintweitii (Huth) Munz in J. Arnold Arbor. 48: 191. 1967a. Type: Pakistan. Gilgit-Baltistan: “Bálti. Environs of Skárdo”, 6 Aug.–4 Sep. 1856, leg. A. Schlagintweit 821 (holotype: G [G00390260 image!]; isotypes: B [destroyed], BM [BM000553907 image!], MEL [MEL2407606 image!], US [US00409758 image!]).

##### Notes.

Huth based his description solely on the duplicate in the Barbey herbarium (now G).

#### 45. Delphinium sclerocladum

Taxon classificationPlantaeRanunculalesRanunculaceae

Boiss., Diagn. Pl. Orient. 8: 8. 1849 [basionym].

DFB67C47-CA11-5B0F-BE23-D39897C10B32

Delphinium anthoroideum Boiss. var. sclerocladum (Boiss.) Boiss. Fl. Orient. 1: 85. 1867.
≡ Consolida scleroclada (Boiss.) Schrödinger in Ann. K. K. Naturhist. Hofmus. 27: 44. 1913.
≡ Aconitella scleroclada (Boiss.) Soják in Folia Geobot. Phytotax. Bohem. 4: 448. 1969.
≡ Aconitopsis scleroclada (Boiss.) Kem.-Nath. in Trudy Tbilissk. Bot. Inst. 7: 127. 1940. Type: Syria. Latakia: “montagnes de Latakieh”, May-Jul. 1846, leg. P. E. Boissier s.n. (lectotype, designated here: G-BOIS [G00788332 image!, 3 sheets]; isolectotypes: E [E00438707 image! =photo of G-DC], G-DC, P [P00195757!, P00195758!]).

##### Notes.

Boissier annotated P00195758. Therefore the lectotypification is justified. The G-DC specimen (observed only on the photo at E) bears the date “Jun 1846”. A specimen at JE (JE00018613) could probably be part of the type material.

**45.1. DelphiniumsclerocladumBoiss.var.sclerocladum**

#### 45.2. Delphinium sclerocladum Boiss. var. rigidum

Taxon classificationPlantaeRanunculalesRanunculaceae

(Freyn & Sint.) Hossain & P.H.Davis in Notes Roy. Bot. Gard. Edinburgh 22: 414. 1958.

B07F500D-3124-565E-A81B-0C33256E359D

≡ Delphinium anthoroideum Boiss. var. rigidum Freyn & Sint. in Österr. Bot. Z. 41: 363. 1891 [basionym].
≡ Consolida scleroclada (Boiss.) Schrödinger var. rigida (Freyn & Sint.) P.H.Davis, Fl. Turkey 1: 123. 1965.
≡ Consolida euphratica Schrödinger in Ann. K. K. Naturhist. Hofmus. 27: 43. 1913. Type: Turkey. “Chama ad Euphratem. Ichtik prope Tuzla”, 15 Jul. 1890, leg. P. E. E. Sintenis 2969 (lectotype, designated here: P [P00195794!] (Fig. 2C); isolectotype: LD [LD1011461 image!]).

##### Notes.

Contrary to the quotation of Hossain and Davis in their protologue, the type specimen was found neither at W nor at WU (probably destroyed during WWII). At JE, the specimen *Sintenis 2969* (JE00018614) does not correspond to type material since it bears the mention “Erzinghan: in declivibus ad Euphratem prop. Sürek” with the date 17 Jul. 1890. In 1913, Schrödinger recombined the varietal rank “*rigidum*” by renaming it as *Consolidaeuphratica*. He quoted as synonym D.anthoroideumvar.rigidum Freyn & Sint. In order to resolve both names simultaneously, we designate the specimen “*Sintenis 2969*” at P as lectotype of D.anthoroideumvar.rigidum Freyn & Sint. and *C.euphratica* Schrödinger.

#### 46. Delphinium songoricum

Taxon classificationPlantaeRanunculalesRanunculaceae

(Kar. & Kir.) Nevski, in Komarov, V. L., Fl. URSS 7: 109. 1937.

5FDBFB66-5007-55A6-8CC0-38B0892B3106

≡ Delphinium camptocarpum Fisch. & C.A.Mey. var. songoricum Kar. & Kir. in Bull. Soc. Nat. Mosc. 15:136. 1842 [basionym].
≡ Consolida songorica (Kar. & Kir.) Nevski in Komarov, V. L., Fl. URSS 7: 109. 1937. Type: Russia. “In arenosis Songoriae ad fl. Lepsa”, 1841, leg. G. S. Karelin & I. P. Kirilow 1165 (holotype: LE, not found; isotypes: BM [BM000946032 image!], H [H1252673 image!], K [K001394824 image!], NY [NY00353417 image!], P [P00197046!, P00197047!]).

##### Notes.

The holotype should be at LE (indicated by Nevski 1937), although we did not find it.

#### 47. Delphinium staminosum

Taxon classificationPlantaeRanunculalesRanunculaceae

(P.H.Davis & Sorger) Jabbour in Global Fl. 4(1): 73. 2018.

F75643D4-5A20-5055-A983-921EF7143703

≡ Consolida staminosa P.H.Davis & Sorger in Notes Roy. Bot. Gard. Edinburgh 40: 89. 1982 [basionym]. Type: Turkey. Niğde: “Çaykavak pass”, 19 Jul. 1979, leg. Hübl, Meusel & Valant 7-19-13 (holotype: E [not found]; isotype: LI [LI02796901 image!]).

##### Notes.

Despite investigations by the curators, the specimen at E was not found, and contrary to what Davis and Sorger indicated in their protologue, duplicates were found neither at WU nor at W.

#### 48. Delphinium stapfianum

Taxon classificationPlantaeRanunculalesRanunculaceae

(P.H.Davis & Sorger) Jabbour in Global Fl. 4(1): 73. 2018.

508F777D-25D3-5007-9D2B-18D3299B427F

≡ Consolida stapfiana P.H.Davis & Sorger in Notes Roy. Bot. Gard. Edinburgh 40: 89. 1982 [basionym]. Type: Turkey. Antalya: “20 km SW of Korkuteli, 1200 m, field margins”, 12 Jul. 1968, leg. F. Sorger 68-27-11 (holotype: E, not found; isotype: LI [LI02796918 image!]).

##### Notes.

Despite investigations by the curators, the specimen at E was not found.

#### 49. Delphinium stenocarpum

Taxon classificationPlantaeRanunculalesRanunculaceae

Hossain & P.H.Davis in Notes Roy. Bot. Gard. Edinburgh 22: 413. 1958 [basionym].

B2BC30D1-4DE5-5F87-8665-03795B92442D

≡ Consolida stenocarpa (Hossain & P.H.Davis) P. H. Davis in Notes Roy. Bot. Gard. Edinburgh 26: 173. 1965.
≡ Aconitella stenocarpa (Hossain & P.H.Davis) Soják in Folia Geobot. Phytotax. Bohem. 4: 448. 1969. Type: Turkey. Konya: “between Ağabeyli and Körkuyu”, 8 Sep. 1949, leg. P. H. Davis 16638 (holotype: E [E00438702 image!]; isotypes: K [K000692450 image!, K000692449 image!]).

##### Notes.

Hossain and Davis formally designated the holotype of *Delphiniumstenocarpum* at E, where we found E00438702 annotated by themselves.

#### 50. Delphinium stocksianum

Taxon classificationPlantaeRanunculalesRanunculaceae

Boiss., Diagn. Pl. Orient., Ser. 2, 1: 12. 1853 [basionym].

65464159-807D-5C16-B94E-B824FD8A6BCE

≡ Consolida stocksiana Nevski in Komarov, V. L., Fl. URSS 7: 111. 1937. Type: Pakistan. “Baluchistan”, 1851, leg. J. E. Stocks 979 (holotype: G-BOIS [G00789496 image!, 3 sheets]; isotypes: K [K000075584 image!, K000075586 image!, K000075587 image!]).

##### Notes.

The holotype is a folder of three sheets.

#### 51. Delphinium sulphureum

Taxon classificationPlantaeRanunculalesRanunculaceae

Boiss. & Hausskn. in Boiss., Fl. Orient. 1: 81. 1867 [basionym].

B9787643-8334-528E-9E8C-09240F161BA1

≡ Consolida sulphurea (Boiss. & Hausskn.) P.H.Davis in Notes Roy. Bot. Gard. Edinburgh 26: 175. 1965. Type: Turkey. Maraş: “Montes azia/ Marasch”, 1865, leg. H. C. Haussknecht s.n. (holotype: G-BOIS [G00788353 image!, 2 sheets]; isotype: P [P00201052!]).

##### Notes.

In the protologue, Boissier quoted an unnumbered Haussknecht’s collection with the following indication “in graminosis montium Syriae borealis prope Marasch alt. 4000’”. Davis (1965) indicated as “types” a specimen housed at K. However, Boissier did not examine this specimen and based his description solely on specimens in his herbarium, where we found a collection folder containing two sheets. One bears a printed label of Haussknecht’s collection with his handwritten additions of locality “In apris v. Karabigukle et pr. Marasch”, and the date “Aug. 65”. The other one bears three of Boissier’s handwritten labels, one of which bears the mention of the locality “Montes azia/ Marasch” and the date “1865”.

At JE, K, and P, we found Haussknecht’s duplicates (JE00018589, K000692378, and P00201052) with a label handwritten by himself bearing the locality “Uffoschikle” (or “Uffoschakli”) with the date 11 July 1865. A duplicate at JE bears, in addition, the number 970. All these specimens correspond to syntypes.

#### 52. Delphinium teheranicum

Taxon classificationPlantaeRanunculalesRanunculaceae

Boiss., Fl. Orient. 1: 85. 1867 [basionym].

9D780038-423D-5B9F-B18B-DFC4A363A376

≡ Consolida teheranica (Boiss.) Rech. f. in Ann. Nat. Mus. Wien 51: 376. 1940.
≡ Aconitella teheranica (Boiss.) Soják in Folia Geobot. Phytotax. Bohem. 4: 448. 1969.
≡ Aconitopsis teheranica (Boiss.) Kem.-Nath. in Trudy Tbilissk. Bot. Inst. 7: 127 1940. Type: Iran. “Teheran”, leg. C. G. T. Kotschy, s.n. (lectotype, designated here: G-BOIS [G00788331 image!]).

##### Notes.

In the protologue, Boissier (1867) based the species description on the specimen *Kotschy 884* housed at W (“in herb. Mus. Vindob!”). Iranshahr (1992) indicated to have seen this specimen at G. However, after investigation, the specimen *Kotschy 884* was found neither at W, nor at WU, nor G. In G-BOIS, we found a Kotschy’s gathering of *D.teheranicum* with a fragmentary specimen (probably a part of the specimen cited in the protologue) and a label written by Boissier. We designate this specimen as the lectotype.

#### 53. Delphinium tenuissimum

Taxon classificationPlantaeRanunculalesRanunculaceae

Sm., Fl. Graec. Prodr. 1: 370. 1809 [basionym].

281CA805-AE99-5CB6-8C73-F1AFC33384B2

≡ Consolida tenuissima (Sm.) Soó in Österr. Bot. Z. 71: 241. 1922. Type: Greece. Sterea Ellas: “Mt Hymethus”, s.d., leg. J. Sibthorp s.n. (holotype: OXF [Sib-1234 image!], isotype: BM [BM000613696 image!]).

##### Notes.

Sibthorp’s herbarium is housed at OXF, where we found a duplicate bearing the locality annotation “Mt Hymethus”.

#### 54. Delphinium thirkeanum

Taxon classificationPlantaeRanunculalesRanunculaceae

Boiss., Fl. Orient. 1: 84. 1867 [basionym].

DA0907BC-5348-5AEE-95AE-68FD968996FF

≡ Consolida thirkeana (Boiss.) Schrödinger in Abh. K. K. Zool.-Bot. Ges. Wien 4(5): 62. 1909.
≡ Aconitella thirkeana (Boiss.) Soják in Folia Geobot. Phytotax. Bohem. 4: 448. 1969.
≡ Aconitopsis thirkeana (Boiss.) Kem.-Nath. in Trudy Tbilissk. Bot. Inst. 7: 125. 1940. Type: Turkey. “Amasia et Tokat”, s.d., F. Wiedemann s.n. (lectotype, designated here: G-BOIS [G00788335 image!], isolectotype: E [E00438704 image! =photo of G-BOIS]).

##### Notes.

In his protologue, Boissier (1867) cited two gatherings: “in Bithynia, Thirke” and “circa Amasia et Tokat Anatoliae, Wiedem.”. Wiedemann’s gathering at G-BOIS is here chosen as lectotype. Munz (1967a) indicated that he saw a duplicate of this gathering at GH and K. However, the duplicate at GH could not be found by the curator. It is unclear whether the Wiedemann’s gathering at K of *D.thirkeanum* (K000075588), which is apparently a duplicate from a specimen at LE, belongs to the type material. Moreover, Huth (1895) quotes a specimen at LE, which was not observed.

#### 55. Delphinium tomentosum

Taxon classificationPlantaeRanunculalesRanunculaceae

Boiss. in Ann. Sci. Nat. Bot. Ser. 2, 16: 365. 1841 [basionym].

3EB902E6-A55E-5FB9-8074-6DB70C65A9AC

≡ Consolida tomentosa (Boiss.) Schrödinger in Abh. K. K. Zool.-Bot. Ges. Wien 4(5): 62. 1909. Type: Syria. “Persia”, 1836, P. M. R. Aucher-Eloy 77 (lectotype, designated here: P [P00201127!] (Fig. 2D)).

##### Notes.

In his protologue, Boissier cited two gatherings of Aucher-Eloy: “N.76. Alep” and “77. Persia”. Chowdhuri et al. (1958. 22: 418) erred in designating as lectotype the gathering *Aucher 75* “Syria!” that Boissier (1841) indicated under *Delphiniumvirgatum* Poir. (in fact, *Aucher 75* corresponds to *D.peregrinum* L.). No sheet of *Aucher 76* bears any annotation from Boissier, whereas P00201127 (*Aucher 77*) is annotated. We designate this latter specimen as the lectotype. No duplicate was found.

#### 56. Delphinium trigonelloides

Taxon classificationPlantaeRanunculalesRanunculaceae

Boiss. in Ann. Sci. Nat. Bot. Ser. 2, 16: 366. 1841 [basionym].

CEB0B00E-3A5E-5F52-B1E1-76B6CBD618DD

≡ Consolida trigonelloides (Boiss.) Munz in J. Arnold Arbor. 48: 190. 1967a. Type: Iran. “Pers. australi”, s.d., leg. P. M. R. Aucher-Eloy 4033 (holotype: G-BOIS [G00788304 image!]; isotypes: BM [BM000570953 image!], G [G00440760 image!, G00440761 image!], K [K000692375 image!], P[P00201193!, P00201194!]).

##### Notes.

Boissier based his species description on the duplicate of his herbarium.

#### 57. Delphinium tuntasianum

Taxon classificationPlantaeRanunculalesRanunculaceae

Halácsy in Magyar Bot. Lapok 11: 117. 1912 [basionym].

BE3C3791-B43B-5D81-82BD-E9C7FEAB0220

≡ Consolida tuntasiana (Halácsy) Soó in Österr. Bot. Z. 71: 239. 1922. Type: Greece. Sterea Ellas: “in regione abietina m. Gerania Megarae”, 23–28 Jun. 1910, B. Tuntas 1245 (lectotype, designated here: WU [WU 01067863 image!, specimen on the bottom left corner]).

##### Notes.

Halácsy based his description of *D.tuntasianum* on the specimen *Tuntas 1245* from the “Plantae exsiccatae Florae Hellenicae” collection. Three Tuntas specimens of *D.tuntasianum*, each one including several plant individuals, were found at WU: *Tuntas 1245*, *Tuntas 800*, and *Tuntas*s.n. Examination of *Tuntas 1245* shows two different dates: “23–28 Jun. 1910” (printed) and “10/23 Mai 1911” (handwritten), this later date being the same as the gathering “s.n.”. Therefore, the gathering “1245” is probably a combination of two different collections. We designate it as a lectotype despite the uncertainty regarding its collection date.

#### 58. Delphinium uechtritzianum

Taxon classificationPlantaeRanunculalesRanunculaceae

Huth in Bot. Jahrb. Syst. 20: 378. 1895 [basionym].

4EDE620E-B0C3-59DD-BE49-8C76F738F7BE

≡ Consolida uechtritziana (Huth) Soó in Österr. Bot. Z. 71: 236. 1922. Type: Albania. “In arvis ad Zojz”, 1889, leg. A. Baldacci s.n. (lectotype, designated here: G [G00414314 image!, 2 sheets]).

##### Notes.

In his protologue, Huth quotes two syntypes (*Pančic 1881* seen in three different herbaria, and *Baldacci 1889* seen in the Barbey herbarium, now G). At G, there is a folder with two sheets of the second gathering that we designate here as lectotype.

## Discussion and conclusions

According to the nomenclatural revision presented here, Delphiniumsubg.Consolida consists of 58 species. This work will facilitate a taxonomic study aimed at revising the circumscription of sections within D.subg.Consolida. Infrasubgeneric relationships were tackled and discussed in Jabbour and Renner (2011). Moreover, a thorough taxonomic study of the species-rich subgenera *Delphinastrum* (DC.) Peterm. and *Oligophyllon* Dimitrova is now timely, as they are the last subgenera in *Delphinium* that still require revision.

## Supplementary Material

XML Treatment for Delphinium subg. Consolida

XML Treatment for Delphinium aconiti

XML Treatment for Delphinium ajacis

XML Treatment for Delphinium anthoroideum

XML Treatment for Delphinium arenarium

XML Treatment for Delphinium armeniacum

XML Treatment for Delphinium aucheri

XML Treatment for Delphinium axilliflorum

XML Treatment for Delphinium baluchistanicum

XML Treatment for Delphinium barbatum

XML Treatment for Delphinium brevicorne

XML Treatment for Delphinium camptocarpum

XML Treatment for Delphinium coelesyriacum

XML Treatment for Delphinium consolida

XML Treatment for Delphinium consolida L. subsp. paniculatum

XML Treatment for Delphinium consolida L. subsp. divaricatum

XML Treatment for Delphinium cornutum

XML Treatment for Delphinium cruciatum

XML Treatment for Delphinium deserti-syriaci

XML Treatment for Delphinium flavum

XML Treatment for Delphinium glandulosum

XML Treatment for Delphinium gombaultii

XML Treatment for Delphinium halophilum

XML Treatment for Delphinium hellesponticum

XML Treatment for Delphinium hispanicum

XML Treatment for Delphinium hohenackeri

XML Treatment for Delphinium incanum

XML Treatment for Delphinium intrincatum

XML Treatment for Delphinium kabulianum

XML Treatment for Delphinium kandaharicum

XML Treatment for Delphinium leptocarpum

XML Treatment for Delphinium linarioides

XML Treatment for Delphinium lineolatum

XML Treatment for Delphinium lorestanicum

XML Treatment for Delphinium mauritanicum

XML Treatment for Delphinium oliverianum

XML Treatment for Delphinium olopetalum

XML Treatment for Delphinium paradoxum

XML Treatment for Delphinium persicum

XML Treatment for Delphinium phrygium

XML Treatment for Delphinium phrygium Boiss. subsp. thessalonicum

XML Treatment for Delphinium pubescens

XML Treatment for Delphinium pusillum

XML Treatment for Delphinium raveyi

XML Treatment for Delphinium rugulosum

XML Treatment for Delphinium saccatum

XML Treatment for Delphinium samium

XML Treatment for Delphinium schlagintweitii

XML Treatment for Delphinium sclerocladum

XML Treatment for Delphinium sclerocladum Boiss. var. rigidum

XML Treatment for Delphinium songoricum

XML Treatment for Delphinium staminosum

XML Treatment for Delphinium stapfianum

XML Treatment for Delphinium stenocarpum

XML Treatment for Delphinium stocksianum

XML Treatment for Delphinium sulphureum

XML Treatment for Delphinium teheranicum

XML Treatment for Delphinium tenuissimum

XML Treatment for Delphinium thirkeanum

XML Treatment for Delphinium tomentosum

XML Treatment for Delphinium trigonelloides

XML Treatment for Delphinium tuntasianum

XML Treatment for Delphinium uechtritzianum
